# Insights into the expanding intestinal phenotypic spectrum of SOCS1 haploinsufficiency and therapeutic options

**DOI:** 10.1007/s10875-023-01495-7

**Published:** 2023-05-09

**Authors:** Marco M. Rodari, Dominique Cazals-Hatem, Mathieu Uzzan, Nicolas Martin Silva, Anis Khiat, Minh Chau Ta, Ludovic Lhermitte, Aurore Touzart, Sylvain Hanein, Cléa Rouillon, Francisca Joly, Adrienne Elmorjani, Julie Steffann, Nadine Cerf-Bensussan, Marianna Parlato, Fabienne Charbit-Henrion

**Affiliations:** 1 Université Paris-Cité, Institut Imagine, Laboratory of Intestinal Immunity, INSERM U1163, Paris, France; 2 Department of Pathology, Beaujon Hospital, Assistance Publique-Hôpitaux de Paris, Clichy, France; 3 Department of Gastroenterology, IBD unit, Beaujon Hospital, Assistance Publique-Hôpitaux de Paris, Clichy, France; 4 Paris Est Créteil University UPEC, Assistance Publique-Hôpitaux de Paris (AP-HP), Henri Mondor Hospital, Gastroenterology department, Fédération Hospitalo-Universitaire TRUE InnovaTive theRapy for immUne disordErs, F-94010 Créteil, France; 5 Department of Internal Medicine, Caen University Hospital, Caen, France; 6 Université Paris Cité, Institut Necker Enfants-Malades INEM, Institut National de La Santé Et de La Recherche Médicale (Inserm), U1151, Paris, France; 7 Laboratory of Hematology, Assistance Publique-Hôpitaux de Paris, Hôpital Necker Enfants-Malades 75743, Paris, France; 8 Bioinformatic Platform, Institute of Genetic Diseases, INSERM UMR1163, Imagine, Université Paris-Cité and Structure Fédérative de Recherche Necker, 75015 Paris, France; 9 Department of Gastroenterology, Caen University Hospital, Caen, France; 10 Genomic Medecine of Rare Diseases, Hôpital Necker-Enfants Malades, Assistance Publique-Hôpitaux de Paris, Paris, France

**Keywords:** Autoimmune enteropathy, JAK inhibition, JAK-STAT, SOCS1 haploinsufficiency

## Abstract

**Purpose:**

Hyper activation of the JAK-STAT signaling underlies the pathophysiology of many human immune–mediated diseases. Herein, the study of 2 adult patients with SOCS1 haploinsufficiency illustrates the severe and pleomorphic consequences of its impaired regulation in the intestinal tract.

**Methods:**

Two unrelated adult patients presented with gastrointestinal manifestations, one with Crohn’s disease-like ileo-colic inflammation refractory to anti-TNF and the other with lymphocytic leiomyositis causing severe chronic intestinal pseudo-occlusion. Next-generation sequencing was used to identify the underlying monogenic defect. One patient received anti-IL-12/IL-23 treatment while the other received the JAK1 inhibitor, ruxolitinib. Peripheral blood, intestinal tissues, and serum samples were analyzed before-and-after JAK1 inhibitor therapy using mass cytometry, histology, transcriptomic, and Olink assay.

**Results:**

Novel germline loss-of-function variants in *SOCS1* were identified in both patients. The patient with Crohn-like disease achieved clinical remission with anti-IL-12/IL-23 treatment. In the second patient with lymphocytic leiomyositis, ruxolitinib induced rapid resolution of the obstructive symptoms, significant decrease of the CD8+ T lymphocyte muscular infiltrate, and normalization of serum and intestinal cytokines. Decreased frequencies of circulating Treg cells, MAIT cells, and NK cells, with altered CD56^bright^:CD16^lo^:CD16^hi^ NK subtype ratios were not modified by ruxolitinib.

**Conclusion:**

SOCS1 haploinsufficiency can result in a broad spectrum of intestinal manifestations and need to be considered as differential diagnosis in cases of severe treatment-refractory enteropathies, including the rare condition of lymphocytic leiomyositis. This provides the rationale for genetic screening and considering JAK inhibitors in such cases.

**Supplementary Information:**

The online version contains supplementary material available at 10.1007/s10875-023-01495-7.

## Introduction

Deciphering the cellular and molecular mechanisms driving intestinal inflammatory disorders is a crucial step to identify pertinent therapeutic targets. The finding that IBD susceptibility loci are enriched in genes participating in JAK-STAT pathway [1], and the analysis of colitis mouse models highlighting the pro-inflammatory role of several upstream cytokines such as IFN-γ, IL-12, and IL-23 [2] have encouraged the use of antibodies blocking the p40 subunit common to IL-12 and IL-23, as well as recently, of JAK inhibitors. Yet, the use of these drugs remains largely empirical in human IBD. In contrast, rare Mendelian forms of severe inflammatory enteropathies, caused by genetic variants which selectively enhance the JAK-STAT signaling axis, provide unique opportunities to delineate how excessive JAK-STAT signaling may contribute to intestinal inflammation in humans. This is notably the case of germline gain-of-function (GOF) variants in *STAT3* [3–5], *STAT1* [6], *JAK1* [7], and somatic variants in *STAT5B* [8] or of loss-of-function (LOF) variants in key negative regulators of the pathway such as *PTPN2* or *SOCS1* [9, 10], all of which have been associated with celiac-like enteropathy, lymphocytic colitis, and more rarely Crohn-like inflammation. These human diseases also represent privileged situations to validate the therapeutic benefit of drugs targeting the JAK-STAT pathway. We and others have reported that JAK inhibitors can resolve or improve intestinal inflammation in patients with GOF variants in *JAK1*, *STAT1*, or *STAT3* [4, 5, 7, 11, 12]. Recent reports have identified *SOCS1* haploinsufficiency (HI) as a possible cause of systemic autoimmunity associated in one case with celiac-like disease [9, 13–16]. Here, the study of two unrelated patients allows to expand the intestinal phenotype spectrum of *SOCS1* HI and illustrates the broad range of intestinal inflammatory manifestations that can be induced by hyperactivation of the JAK-STAT pathway. Our observations also highlight how genetic diagnostic can sort out molecular mechanisms underlying unusual phenotype or resistance to treatment and guide therapy.

## Material and Methods

### Targeting Next-generation and Sanger Sequencing

Custom-targeted next-generation sequencing (TNGS) was performed on genomic DNA extracted from P1’s and P2’s peripheral blood mononuclear cells (PBMC), targeting genes previously associated with monogenic enteropathies [17]. In brief, genomic DNA libraries were captured by hybridization using Agilent Sure Select complementary 120-pb probes to cover all exons of the selected genes. SOCS1 variants were confirmed by Sanger sequencing using the primers: *socs1*_For-cccagctcacctctttgtct; *socs1*_Rev-cacatggttccaggcaagta.

### Site-directed Mutagenesis

The WT human SOCS1 (NM_003745) cDNA, subcloned into pCMV-HA from Clontech (Cat# 635690), served to generate SOCS1 variants using GENEART® Site-Directed Mutagenesis System (Invitrogen, ThermoFisher Scientific) with the following primers: *socs1*_c.298_301dup_For-agcccgtgggcacctaccttcctggtgcgcgaca, *socs1*_c.298_301dup_Rev-tgtcgcgcaccaggaaggtaggtgcccacgggct; *socs1*_c.58C>T_FW-acagcagcagagccctgacggcggccagaac, *socs1*_c.58C>T_RV-gttctggccgccgtcagggctctgctgctgt. All variant SOCS1 plasmids were confirmed by Sanger sequencing.

### Cell Culture, Transfection, and Immunoblotting

The Lenti-X™ 293T Cell Line (Clontech) was cultured at 37°C in DMEM GLUTAMAX (Invitrogen) containing 10% FCS, and penicillin and streptomycin (100 U/ml each; Invitrogen). Lenti-X™ 293T cells were seeded in T-75 flask and cultured for 16 to 24 h before transfection using lipofectamine 2000 (Life Technologies) and with 10 μg of empty vector, WT-SOCS1 and SOCS1-mutant plasmids. The pPURO-FLAG-HA-EGFP (6104bp) plasmid served as transfection control. 18 h post-transfection, cells were harvested after washing with ice-cold PBS and then lysed with ice-cold RIPA buffer supplemented with protease inhibitors (Roche). For STAT1/STAT5 phosphorylation assay, Epstein–Barr-virus (EBV)–transformed B lymphoblastoid cell lines (EBV-LCL cells) were generated from PBMCs obtained from P2 and control (Ctrl), and then cultured in RPMI 1640 (Life Technologies) supplemented with 10% fetal bovine serum (FBS) and penicillin/streptomycin (100 U/ml each; Invitrogen). EBV-LCL cells were stimulated with 10^3^ IU/ml IFN-γ or 10^4^ IU/ml IL-2 for 30 min with or without Ruxolitinib (200 μM).

Cell lysates were separated by 4−15% SDS-PAGE gels (Bio-Rad Laboratories), then transferred to polyvinylidene difluoride membranes using the Trans-Blot Turbo Blotting System (Bio-Rad Laboratories). After blocking with 5% skim milk for 1 h, membranes were incubated with anti-SOCS1 (A156, #3950 Cell Signaling), anti-HA tag (HA.C5, ab18181, Abcam), anti-STAT1 (9H2, #9176, Cell Signaling), anti-pSTAT-1 (58D6, #9167, Cell Signaling), anti-STAT5 (D206Y, #94205, Cell Signaling), anti-pSTAT5 (C11C5, #9359, Cell Signaling), and anti-GAPDH (14C10, #2118, Cell Signaling) antibodies overnight at 4°C. Membranes were then incubated with anti-mouse (#7076, Cell Signaling) or anti-rabbit (#7074, Cell Signaling) antibodies conjugated with HRP for 1 h at room temperature and visualized using Clarity™ Western ECL Substrate (Bio-Rad) and the ChemiDoc XRS+ imaging system (Bio-Rad).

### Reverse Transcription and PCR

For cytokine mRNA expression studies, RNA was extracted from formalin-fixed paraffin-embedded (FFPE) histological tissues using RNAeasy FFPE kit (Qiagen). Briefly, freshly cut FFPE tissue 5-μm sections were incubated with Qiagen deparaffinization solution prior to RNA purification. Following proteinase K tissue digestion and subsequent incubation at 56°C and 80°C, RNAeasy MinElute spin columns were used to elute RNA, according to the manufacturer’s instructions. Quantitative reverse transcriptase PCR was performed on C1000 Touch Thermal Cycler apparatus (Bio-Rad) using TaqMan universal PCR Master Mix and Taqman probe assays (ThermoFisher Scientific): RPLP0 (Hs99999902_m1); GzmB (Hs01554355_m1); IFNG (Hs99999041_m1); IL10 (Hs00961622_m1); IL12A (Hs009673447_m1); IL21 (Hs00222327_m1). Results were expressed as relative expression 2^–ΔCt^ normalized to the housekeeping ribosomal protein large PO (*RPLPO*) gene.

### Immunohistochemistry

Surgical specimens were obtained from P2 at the time of presentation and then 6 months later after ruxolinitib introduction, fixed in 10% buffered formalin for 24 h, paraffin-embedded, and 3μm paraffin sections were stained with hematoxylin and eosin. Immunophenotyping of the lymphoid infiltrate was performed on Dako Omnis automated immunostainer, for CD3 (Dako, polyclonal), CD8 (Dako, C8/144B clone), and Granzyme B (Dako, GzB-7 clone).

### Proximity Extension Assay on Plasma Samples—OLINK

Inflammatory cytokine levels were determined using a Proximity Extension Assay technology based on cytokine-specific oligonucleotide-labeled antibody probe pairs (Olink Target 48 Cytokine Panels, Olink). Frozen plasma samples were processed by Olink service. For the heatmap, absolute concentration value of each cytokine (pg/mL) was normalized to the median values in age-matched controls and the *x*-fold standard deviation above or below median was plotted for each value. Principal component analysis (PCA) was performed using absolute concentration values.

### Cytometry by Time-of-light (CYTOF)

CyTOF staining was performed using a deep immunephenotyping panel of antibodies (Maxpar Direct Immune Profiling Assay, Fluidigm) according to the manufacturer’s instructions. Briefly, following Ficoll-Paque^TM^ PLUS (GE Healthcare) density gradient centrifugation, 5 × 10^6^ PBMCs were washed in Maxpar Staining Buffer (Fluidigm) and incubated for 30 min at room temperature with metal-tagged surface antibodies and Rh103 live/dead indicator. Finally, the samples were washed and fixed with 1.6% formaldehyde and their DNA labeled with a 125-nM intercalation solution obtained adding Cell-ID Intercalator into Maxpar Fix and Perm Buffer (Fluidigm). Cells were incubated overnight at 4°C and conserved at −80°C until ready for acquisition. Samples were acquired with a CyTOF2 Helios mass cytometer (Fluidigm). Data, exported as a flow cytometry file (FCS), were analyzed using FlowJo™ v10.8 software (BD Life Sciences) and OMIQ data analysis software (www.omiq.ai).

### Flow Cytometry

PBMCs from healthy controls and P2 were washed and stained with FITC CD4 (SK3), APC TCR Vα7.2 (3C10), PerCP/Cy5.5 CD8 (SK1), PE/Cy7 CD3 (SK7), APC/Cy7 CD14 (HCD14), APC/Cy7 CD19 (SJ25C1), Brilliant Violet 421 CD161 (HP-3G10), all antibodies from SONY and LIVE/DEAD Fixable Near-IR Dead Cell Stain Kit (Invitrogen) for 20 min at 4°C. Cells were washed in PBS 2% human serum AB (Sigma). Flow data were acquired with a BD FACSCanto™ II and analyzed with FlowJo™ v10.8 software (BD Life Sciences).

### Statistical Analysis

Data analysis and visualization were performed using GraphPad Prism v. 9.5.0 (730).

## Results

### Patients’ Description

**Fig. 1:**
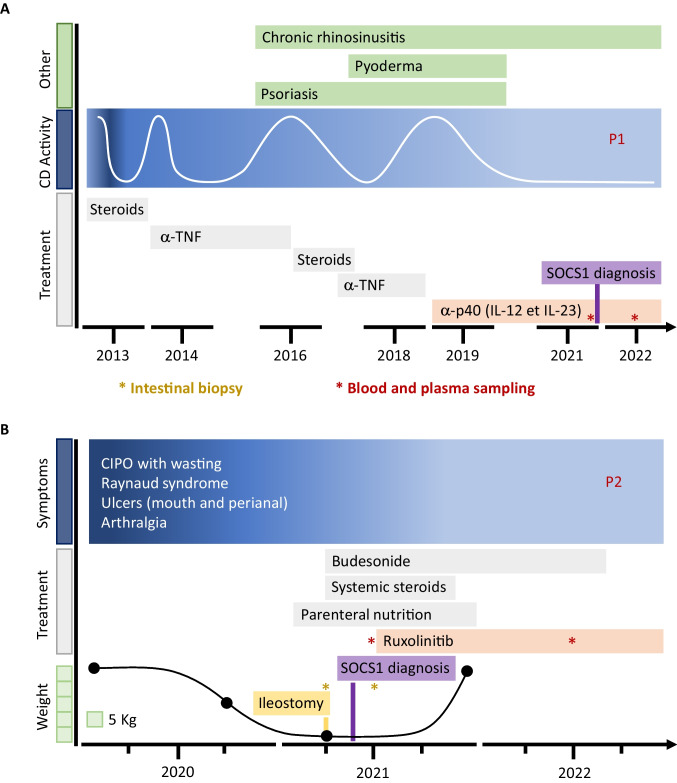
Disease timeline and treatment strategies. **A** P1 was initially treated by infliximab (anti-TNF-α) and after relapse by ustekinumab (anti-IL-12/IL-23). **B** P2 was initially treated with systemic steroids. Ruxolitinib was introduced after SOCS1 diagnosis. A single asterisk (*) indicates a blood sampling before and after 1 year of treatment

P1 (family 1), a 28-year-old woman born from non-consanguineous parents, developed ileo-colic chronic inflammation resembling Crohn’s disease at the age of 18. Her medical history included repeated infections in infancy, i.e., otitis and sinusitis, until the age of 7. Her inflammatory bowel disease did not respond to steroids and azathioprine but was improved by adalimumab. P1 developed, however, psoriasis and pyoderma gangrenosum at the age of 21 with slow resolution and severe destructive and necrotic chronic rhinosinusitis without nasal polyps at the age of 22. Laboratory tests ruled out an underlying primary immune deficiency, especially a primary antibody production failure. She relapsed under adalimumab and was started on ustekinumab at the age of 24 with complete resolution of intestinal symptoms. Repeated control ileo-colonoscopy and biopsies were normal. She still has chronic rhinosinusitis with a significant improvement under cotrimoxazole started at her 26 years (Fig. 1A, Table 1).

**Table 1:** Clinical features of SOCS1 haploinsufficient patients

	Sex	Age onset (year)	Current age (year)	Extra-digestive symptoms (age onset year)	GI symptoms (age onset year)	Biology	Treatment	Status at last news
**Family 1**								
P1	F	18	28	Sinusitis (childhood) Psoriasis and pyoderma gangrenosum (21, under IFX) Severe chronic rhinosinusitis without nasal polyps (22)	Ileo-colic Crohn’s disease (18)	ANA 1/160 ANCA+: PR3 and MP0 – Antithyroglobulin Ab: IgG, A, M, IgG subclasses, B cells, T cells, NK cells: normal range	Steroid, AZA, IFX no response, improvement of GI symptoms only under ADA Under ustekinumab: in remission for GI symptoms only Improvement of ENT symptoms under cotrimoxazole	GI: in remission ENT: partial improvement
**Family 2**								
I.1	M	30	84	Anosmia (30) Severe asthma and bronchopneumopathies (50)	-	ND	-	Deceased
P2 II.3	F	24	68	Asthma (24) Dry eyes and mouth (40) Bipolar aphtosis (40) Raynaud’s syndrome (38) Severe arthritis (40) Anosmia (52) Méningioma 17mm	CIPO due to lymphocytic leiomyosistis (55–58)	ASCA 29U/ml, ANA 1/80, anti- smooth muscle positive, anti-mitochondria 1/160, all others auto-Ab negative (cf list)	Parenteral nutrition Systemic steroid and Budesonide: discontinued after remission with ruxolitinib	In remission
II.6	M	55	66	Asthma (childhood) Psoriasis (mild) Tuberculosis and aspergillosis (52) Idiopathic pericarditis (65)	Severe dysphagia (55) Mild eosinophilic infiltrate in oesophagus’ biopsies (<20/100)	FAN 1/100 ANCA + ANCA PR3 - ANCA MPO - ASCA -	Proton Pump inhibitor	In remission
III.1	F	Birth	Died at birth	Died at birth of severe epidermolysis bullosa (no genotype)				Deceased
III.3	F	-	38	-	-	-	-	No symptoms
III.5	F	11	36	Asthma (childhood) Sclerodermia (11) Myositis (15) Raynaud syndrome (34)	Chesnut and peanut allergies without anaphylaxis	ND	MTX and steroids between 11and 18 years No TT since 18	In remission
III.7	M	25	39	Severe psoriasis	-	ND	Local steroids	Active disease

*GI* gastrointestinal, *IFX* infliximab, *ADA* adalimumab, *ANA* antinuclear antibodies, *ANCA* antineutrophil cytoplasmic antibodies, *ASCA* anti-*Saccharomyces cerevisiae* antibodies, *AZA* azathioprine, *CIPO* chronic intestinal pseudo-occlusion, *ENT* ear-nose-throat, *MTX* methotrexate, *ND* not done, *TT* treatment

List of negative auto-Ab in P2: anti DNA, HMGCoA reductase, Jo1, Scl70, PL-7, PL-12, EJ, SRP, Mi2, MDA5, TIF1 gamma, Ku, PM/Scl, RNA POL3, Th/To, NOR90, fibrillarin, GP210, Sp100, PML, parietal gastric cells, CCP, cardiolipine, B2GP1, LKM1, LC1, E2PDH, SLA, ANCA, transglutaminase, Ro52, centromere

P2 (family 2), a 61-year-old woman, born from non-consanguineous parents developed severe symptoms of chronic intestinal pseudo-occlusion (CIPO) at the age of 58, leading to a 22-kg weight loss within 1 year (Fig. 1B, Table 1). Her medical history included asthma since the age of 24, severe unclassified peripheral polyarthritis with Raynaud’s syndrome, mouth ulcers, and Sjögren syndrome since the age of 40 and complete anosmia since the age of 52. Her familial history revealed asthma, psoriasis, pericarditis, and dysphagia with mild eosinophilic infiltrate in esophagus’ biopsies in one brother, epidermolysis bullosa responsible for neonatal death in her first daughter, sclerodermia, and myositis starting at the age of 11 in another daughter. Her father, who died at 84, had developed severe asthma and complete anosmia. Because of the major weight loss and obstructive symptoms with vomiting limiting caloric enteral input, parenteral nutrition was started in P2. To alleviate intestinal occlusion, surgical ileostomy was performed. Ileal surgical specimen revealed normal mucosa without villous atrophy (consistent with intestinal and colonic endoscopic biopsies) but massive and diffuse infiltration of muscularis propria by normal-looking small lymphocytes, mainly CD3^+^ CD8^+^ granzyme B^+^ T cells, adjacent to myocyte destruction or atrophy (Fig. 3A). Myenteric plexuses were not-affected (arrow). Analysis of T-cell rearrangements revealed clonal T-cell amplification. Yet, the small size and normal cytological appearance of lymphocytes (Fig. 3A) ruled out lymphoma diagnosis, overall leading to a diagnosis of CIPO due to intestinal lymphocytic leiomyositis, possibly of autoimmune origin.

### SOCS1 Haploinsufficiency Identified in P1 and P2

**Fig. 2:**
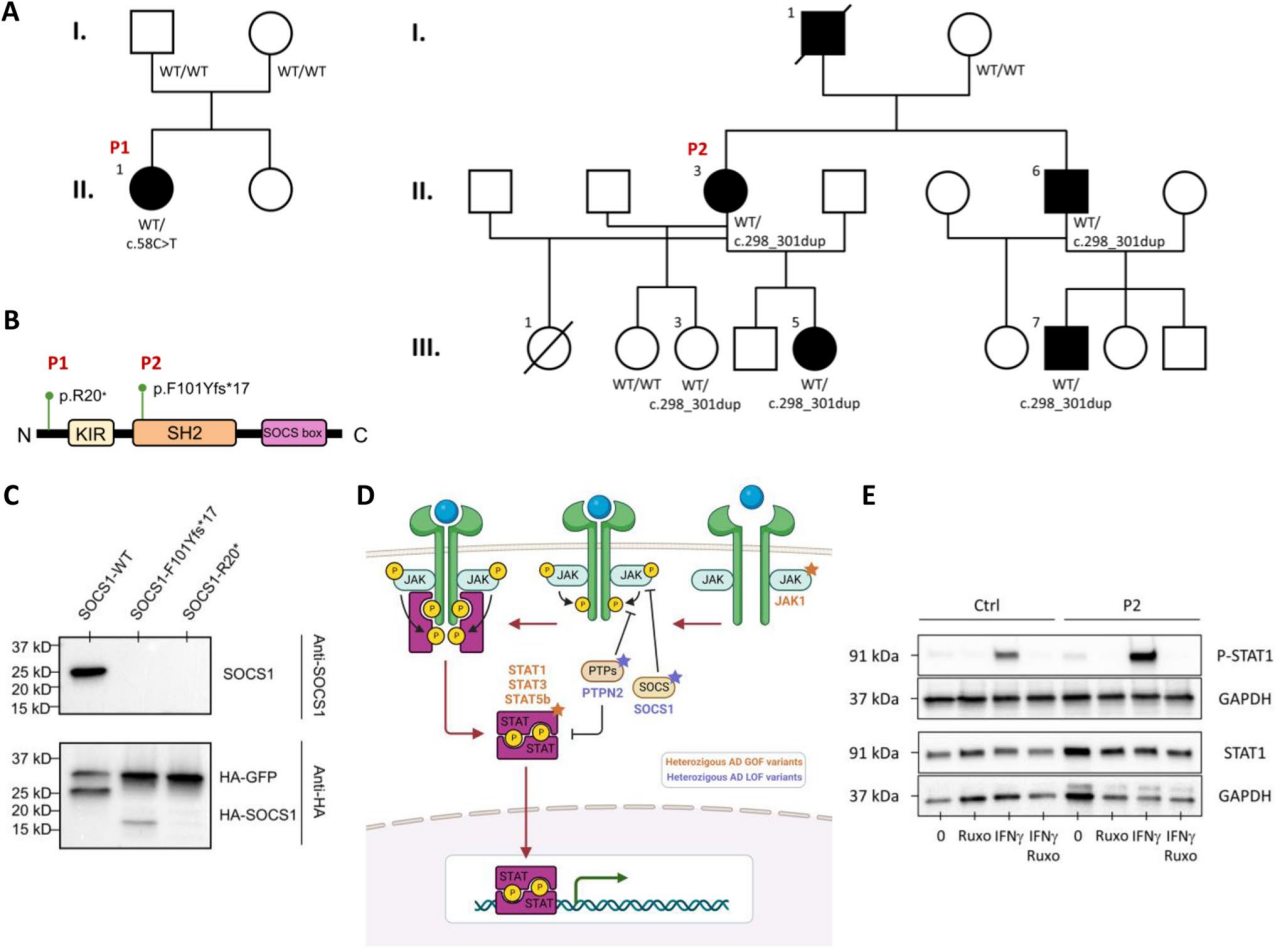
SOCS1 variants in patients with intestinal manifestations. **A** Pedigree showing segregation of heterozygous SOCS1 variants. Affected individuals are represented by a filled circle. **B** Domain structure of SOCS1. **C** SOCS1 expression in HEK293T cells overexpressing SOCS1-WT and mutant alleles. The pPURO-FLAG-HA-EGFP (6104bp) plasmid served as transfection control. **D** Schematic representation of JAK-STAT signaling. A single asterisk (*) indicates members encoded by genes previously associated with intestinal monogenic disorders. **E** p-STAT1 in EBV-LCLs from a healthy control and P1 stimulated with IFN-γ (10^3^ IU/ml for 20 min) with or without 200 μM ruxolitinib. Data are representative of 2 independent experiments

TNGS detected two novel heterozygous variants in *SOCS1*, both predicted to be LOF due to a premature termination codon (c.58C>T/p.Arg20*) and (c.298_301dup/p.Phe101Tyrfs*17) in P1 and P2 respectively (Fig. 2A). Candidate variants were confirmed by Sanger sequencing (Figure S1A) in patients and in family members who were available and/or accepted genetic testing. In family 1, neither parent carried the variant indicating a *de novo* event in P1. In family 2, the *SOCS1* variant was found in P2’s sick brother (IIA) and in her sick daughter (III.7) (see above) as well as in one asymptomatic daughter (III.3), confirming the incomplete penetrance of *SOC1* HI. DNA was not available for P2’s father who had displayed symptoms (see above), but all tested family members carrying the *SOCS1* variant shared the same paternal haplotype, suggesting that the *SOCS1* variant was inherited from him (I.1) (Figure S1B).

The impact of *SOCS1* variants on protein expression was evaluated in Lenti-X™ HEK293T Cell Line by overexpressing the cDNA corresponding to the patient’s mutant alleles. Full-length *SOCS1* was not expressed in P1 but immunoblotting showed a weakly expressed truncated ~15KDa SOCS1 protein in P2, excluding re-initiation of translation (Fig. 2C). *SOCS1* encodes for the inducible suppressors of cytokine signaling 1, part of the negative feedback system of the JAK-STAT pathway in response to activation by interferons or by the γc family of cytokines (Fig. 2D). *SOCS1* features a conserved central Src-homology 2 (SH2) domain (binding to JAKs’ phosphotyrosine-containing sequences), a short C-terminal SOCS box (inducing ubiquitination and proteasomal degradation of captured substrates), and a kinase inhibitory region (KIR domain) (Fig. 2B). Increased level of p-STAT1 and p-STAT5 upon stimulation with IFN-γ and IL-2, respectively, were detected in in patients’ EBV-LCLs compared to control cells (Fig. 2E and Figure S1C), confirming lack of negative feedback due to SOCS1 HI. In addition, aberrant IL-2-induced STAT5 phosphorylation or IFN-γ-induced STAT1 phosphorylation could be controlled in vitro by JAK1/2 inhibitor ruxolitinib (Fig. 2E and Figure S1C).

### CIPO Resolution by Ruxolitinib in Patient 2

**Fig. 3:**
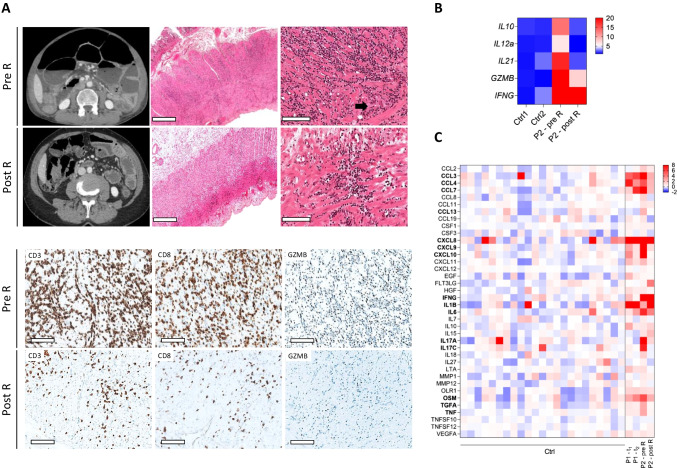
Resolution of P2’s CIPO by ruxolitinib treatment. **A** Image CT scan, histology of lymphocytic intestinal leiomyositis in surgical specimens HES staining (scale bar= 900 μm or 100 μm) and immunohistochemistry staining of CD3^+^, CD8^+^, GzB^+^ lymphocytes before and after 3 months of ruxolitinib (R) (scale bar=100 μm). **B** Quantitative PCR analysis of indicated cytokines in P2’s duodenum before (pre-R) and after 3 months ruxolitinib (post-R). **C** Heatmap of plasma inflammatory cytokines in P2 before (pre-R) and after 1 year ruxolitinib (post-R)

Following histological diagnosis of lymphocytic intestinal leiomyositis on the ileal surgical specimen, P2 was treated with budesonide followed by systemic steroids for a flare of polyarthritis. This treatment improved articular symptoms (albeit with steroid-dependency) but neither CIPO-associated symptoms nor intestinal dilatation. Identification of the *SOCS1* variant led to initiate ruxolitinib 10 mg twice daily, which resulted in spectacular improvement of intestinal symptoms as well as of arthritis, enabling steroids withdrawn. After 3 months, intestinal distension had markedly decreased on the abdominal CT scan (Fig. 3A), allowing ileostomy closure and sampling of an ileal surgical specimen. Its examination revealed persistence of small foci of CD8^+^ T lymphocytes in the longitudinal outer muscular layer, but lymphocyte infiltration had strongly decreased compared with the first surgical biopsy (Fig. 3A). Granzyme B transcripts as well as for IL-12, IL-10, and IL-21 were also decreased further supporting resolution of the inflammatory response (Fig. 3B). After 6 months, P2 had regained 25 kg, weaned from parenteral nutrition and in steroid-free remission for arthritis. At latest news, she was in remission after 18 months on ruxolitinib. Immunophenotyping by mass cytometry was performed on fresh PBMCs from P1 and from P2. No major cell-subtype specific abnormalities were identified but a tendency towards decreased frequencies of circulating Treg cells, MAIT cells, and NK cells, with altered CD56^bright^:CD16^lo^:CD16^hi^ NK subtype ratios, which, however, was not modified by ruxolitinib treatment in P2 (Figure S2). These data are overall in line with previous observations in patients with SOCS1 HI [9]. Finally, cytokines dosage by Olink assay in patients’ plasma revealed an increase in IFN-γ, CCL-3/4/5, CXCL-8/9, IL-6, IL-17, TNF-α, TGF-α, Oncostatin-M in P2 before ruxolitinib that normalized after 1 year of ruxolitinib except for IFN-γ, which was only slightly reduced by this treatment (Fig. 3C, Figure S3).

## Discussion

Here, we report the identification of heterozygous LOF variants in *SOCS1*, a negative regulator of the JAK-STAT signaling pathway, as the cause of refractory intestinal inflammation in two unrelated adult patients with Crohn’s disease-like disease and leiomyositis-related CIPO respectively, both successfully put in remission by targeted therapy.

SOCS1 HI was recently identified as a rare cause of multisystem autoimmunity in a small group of patients presenting notably with systemic lupus erythematosus or autoimmune cytopenia [9, 13–16]. Disease was shown to result from over-activation of the JAK-STAT pathway, a major signaling pathway driving the transcriptional responses downstream numerous inflammatory cytokines. This pathway is tightly regulated by inhibitors, including notably PTPN2 and SOCS1, and haploinsufficiency in either PTPN2 [18] or SOCS1 have been associated with severe autoimmune or inflammatory diseases.

Except for celiac-like disease in one patient [9] and transient diarrhea prior to autoimmune cytopenia in another [13], gastrointestinal (GI) manifestations have not been often reported in SOCS1 deficient patients. A bias in recruitment is plausible. Indeed, we and others have reported GI inflammation, notably affecting the small bowel and manifesting as celiac-like autoimmunity refractory to gluten-free diet, in a substantial fraction of patients with pathogenic variants leading to JAK-STAT hyperactivation, including *STAT3* GOF, *STAT1* GOF, *JAK1* GOF variants, and *PTNP2* HI [4, 5, 10, 19]. The present observations of Crohn’s disease-like ileo-colic chronic inflammation in P1 and CIPO caused by lymphocytic leiomyositis in P2 expands the phenotypic spectrum of SOCS1 HI and further stress the importance to strictly control the JAK-STAT pathway to preserve intestinal homeostasis.

In previous reports of SOCS1 HI, lupus-like manifestations were ascribed to hyperactivation of the JAK-STAT pathway downstream type I or type II interferons [9, 13]. Yet, this pathway can be activated by numerous other cytokines, including IL-12 and IL-23, which are thought to play an important role in Crohn’s disease pathogenesis [2]. In keeping with an important role of these cytokines in P1’s digestive disease and corroborating data in mice showing that negative regulation of IL-12 signaling depends on SOCS1 [20], P1 remission was obtained upon blockade of the P40 subunit common to IL-12 and IL-23 cytokines. In P2, the massive smooth muscle infiltration by clonal expansion of CD8^+^ T cells expressing granzyme B and IFN-γ led us to select the JAK1/2 inhibitor ruxolitinib, which we had previously used successfully to treat autoimmune enteropathy induced by *STAT3* GOF [5, 11], with the goal in mind to block signals from cytokines such as IL-2, IL-15 that are central for CD8^+^ T-cell activation, and IFN-γ that mediates some of CD8^+^ T-cell deleterious effects. Accordingly, ruxolitinib led to resolution of the patient’s intestinal lymphocytic leiomyositis. Of note, targeted therapy failed to rescue the frequency of circulating Treg, MAIT, and NK cells in P2. A longer follow-up may be needed to observe changes in cell frequencies. Yet, we cannot exclude that ruxolitinib may have induced functional phenotypic changes with upregulation of activation markers in NK, and B and T cells as previously shown in STAT1 GOF patients [12]. We and others have previously shown that JAK inhibitors are effective in resolving autoimmune symptoms in patients presenting with STAT GOF phenocopies [4, 5, 7, 11, 12]. In case of severe and progressive disease, one alternative therapeutic choice for this group of patients is the allogeneic hematopoietic stem cell transplantation (HSCT), which however remains associated with significant risk of graft-versus-host disease and an overall poor survival (40 to 60%) [4, 21].

Lymphocytic leiomyositis is a very rare and still enigmatic cause of CIPO, an heterogenous group of congenital or acquired disorders resulting from dysfunction of either intestinal smooth muscle or enteric nervous system [22]. Diagnosis of lymphocytic leiomyositis relies on full-thickness biopsies of the small intestine that are indispensable to demonstrate dense CD8^+^ lymphocyte infiltration of the *muscularis mucosa*. In the few reported cases, disease onset was variable, from infancy to adulthood, sometimes after a gastrointestinal infection, suggesting an environmental trigger. Association of some cases with extra-intestinal autoimmunity led to suggest an autoimmune mechanism and to initiate immunosuppressive treatments, notably by steroids. Yet, steroid efficacy was variable and etiology remained elusive [23]. Our findings provided the first demonstration of an autoimmune mechanism underlying this disease, but they also allowed tailored therapy, permitting rapid remission after failure of steroid treatment. While it remains uncertain if such a precise molecular mechanism will be identified in all cases of lymphocytic leiomyositis, we suggest that the rarity and extreme severity of the disease justifies screening those patients for monogenic disorders predisposing to autoimmunity and notably for gene variants enhancing the JAK-STAT pathway.

More generally, the present findings raise the question of when to initiate genetic testing in adults displaying atypical or treatment-refractory GI manifestations. Causal genetic variants are very rare. Yet, obtaining a molecular diagnosis as early as possible is crucial to guide treatment and to offer the possibility of genetic counseling. Moreover, as we have recently shown in patients with autoimmune enteropathy, identification of a genetic variant was associated with increased and early risk of malignancy, including lymphoma [19], stressing the need of close follow-up. Along this line, the CD8^+^ T-cell expansion infiltrating P2’ muscularis mucosa contained a conspicuous T-cell clone. We suggest, as recently proposed by the British society of Gastroenterology [24], that genomic testing for inflammatory intestinal diseases should be discussed in case of GI manifestations associated with criteria suggestive of an inborn error of immunity, such as increased rate of infections or auto-inflammatory and/or complex autoimmune features. Given increasing evidence that many of these diseases can have a delayed onset, these criteria should also be applied in adult patients.

## Supplementary Information

ESM 1 Figure S1 (A) Sanger-sequencing electropherograms for the affected individuals and carriers. (B) Haplotypes for microsatellite markers are shown in Family 2 and the disease-associated haplotype is boxed in orange. (C) p-STAT5 in EBV-LCLs from a healthy control and P2 stimulated with IL-2 (10^4^ IU/ml for 20 min) with or without Ruxolitinib 200 μM. Data are representative of 2 independent experiments. (PDF 602 kb)

ESM 2 Figure S2 (A-E) Immunophenotype of P1 under ustekinumab treatment, and of P2 before and after 6 months Ruxolitinib treatment by mass cytometry. (F) MAIT cells frequency of P2 before and after 6 months Ruxolitinib treatment by flow cytometry. (PDF 517 kb)

ESM 3 Figure S3 Principal component analyses of cytokines in plasma samples from SOCS1 deficient patients and controls. (PDF 214 kb)

## Data Availability

The data underlying this article will be shared on reasonable request to the corresponding author.

## References

[1] Jostins L, Ripke S, Weersma RK, Duerr RH, McGovern DP, Hui KY (2012). Host–microbe interactions have shaped the genetic architecture of inflammatory bowel disease. Nature..

[2] Neurath MF (2014). Cytokines in inflammatory bowel disease. Nat Rev Immunol..

[3] Milner JD, Vogel TP, Forbes L, Ma CA, Stray-Pedersen A, Niemela JE (2015). Early-onset lymphoproliferation and autoimmunity caused by germline STAT3 gain-of-function mutations. Blood..

[4] Leiding JW, Vogel TP, Santarlas VGJ, Mhaskar R, Smith MR, Carisey A (2022). Monogenic early-onset lymphoproliferation and autoimmunity: natural history of STAT3 gain-of-function syndrome. J Allergy Clin Immunol..

[5] Parlato M, Charbit-Henrion F, Abi Nader E, Begue B, Guegan N, Bruneau J (2019). Efficacy of Ruxolitinib therapy in a patient with severe enterocolitis associated with a STAT3 gain-of-function mutation. Gastroenterology..

[6] Uzel G, Sampaio EP, Lawrence MG, Hsu AP, Hackett M, Dorsey MJ (2013). Dominant gain-of-function STAT1 mutations in FOXP3WT IPEX-like syndrome. J Allergy Clin Immunol..

[7] Gruber CN, Calis JJA, Buta S, Evrony G, Martin JC, Uhl SA (2020). Complex autoinflammatory syndrome unveils fundamental principles of JAK1 kinase transcriptional and biochemical function. Immunity..

[8] Ma CA, Xi L, Cauff B, DeZure A, Freeman AF, Hambleton S (2017). Somatic STAT5b gain-of-function mutations in early onset nonclonal eosinophilia, urticaria, dermatitis, and diarrhea. Blood..

[9] Hadjadj J, Castro CN, Tusseau M, Stolzenberg M-C, Mazerolles F, Aladjidi N (2020). Early-onset autoimmunity associated with SOCS1 haploinsufficiency. Nat Commun..

[10] Parlato M, Nian Q, Charbit-Henrion F, Ruemmele FM, Rodrigues-Lima F, Cerf-Bensussan N (2020). Loss-of-Function mutation in PTPN2 causes aberrant activation of JAK signaling via STAT and very early onset intestinal inflammation. Gastroenterology..

[11] Forbes LR, Vogel TP, Cooper MA, Castro-Wagner J, Schussler E, Weinacht KG (2018). Jakinibs for the treatment of immune dysregulation in patients with gain-of-function signal transducer and activator of transcription 1 (STAT1) or STAT3 mutations. J Allergy Clin Immunol..

[12] Borgström EW, Edvinsson M, Pérez LP, Norlin AC, Enoksson SL, Hansen S (2023). Three adult cases of STAT1 gain-of-function with chronic mucocutaneous candidiasis treated with JAK inhibitors. J Clin Immunol..

[13] Lee PY, Platt CD, Weeks S, Grace RF, Maher G, Gauthier K (2020). Immune dysregulation and multisystem inflammatory syndrome in children (MIS-C) in individuals with haploinsufficiency of SOCS1. J Allergy Clin Immunol..

[14] Körholz J, Gabrielyan A, Sowerby JM, Boschann F, Chen L-S, Paul D (2021). One gene, many facets: multiple immune pathway dysregulation in SOCS1 haploinsufficiency. Front Immunol..

[15] Michniacki TF, Walkovich K, DeMeyer L, Saad N, Hannibal M, Basiaga ML (2022). SOCS1 Haploinsufficiency presenting as severe enthesitis, bone marrow hypocellularity, and refractory thrombocytopenia in a pediatric patient with subsequent response to JAK inhibition. J Clin Immunol..

[16] Thaventhiran JED, Lango Allen H, Burren OS, Rae W, Greene D, Staples E (2020). Whole-genome sequencing of a sporadic primary immunodeficiency cohort. Nature..

[17] Charbit-Henrion F, Parlato M, Hanein S, Duclaux-Loras R, Nowak J, Begue B (2018). Diagnostic yield of next-generation sequencing in very early-onset inflammatory bowel diseases: a multicentre study. J Crohn’s Colitis..

[18] Parlato M, Nian Q, Charbit-Henrion F, Ruemmele FM, Rodrigues-Lima F, Cerf-Bensussan N (2020). Loss-of-function mutation in PTPN2 causes aberrant activation of JAK signaling via STAT and very early onset intestinal inflammation. Gastroenterology [Internet]. Gastroenterology..

[19] Charbit-Henrion F, Haas M, Chaussade S, Cellier C, Cerf-Bensussan N, Malamut G (2022). Genetic diagnosis guides treatment of autoimmune enteropathy. Clin Gastroenterol Hepatol..

[20] Eyles JL, Metcalf D, Grusby MJ, Hilton DJ, Starr R (2002). Negative regulation of interleukin-12 signaling by suppressor of cytokine signaling-1. J Biol Chem..

[21] Leiding JW, Okada S, Hagin D, Abinun M, Shcherbina A, Balashov DN (2018). Hematopoietic stem cell transplantation in patients with gain-of-function signal transducer and activator of transcription 1 mutations. J Allergy Clin Immunol..

[22] Connor FL, Di Lorenzo C. Chronic intestinal pseudo-obstruction: assessment and management. Gastroenterology 2006;130(2 Suppl 1):S29–36. 10.1053/j.gastro.2005.06.081 16473068

[23] Haas S, Bindl L, Fischer HP (2005). Autoimmune enteric leiomyositis: a rare cause of chronic intestinal pseudo-obstruction with specific morphological features. Hum Pathol..

[24] Kammermeier J, Lamb CA, Jones KDJ, Anderson CA, Baple EL, Bolton C (2023). Genomic diagnosis and care co-ordination for monogenic inflammatory bowel disease in children and adults: consensus guideline on behalf of the British Society of Gastroenterology and British Society of Paediatric Gastroenterology. Hepatol Nutrition. lancet Gastroenterol Hepatol..

