# Supplementary figures and images for: Insights into the expanding intestinal phenotypic spectrum of SOCS1 haploinsufficiency and therapeutic options
Source: J Clin Immunol. 2023 May 9;43(6):1403–13. doi: 10.1007/s10875-023-01495-7 (PMC10354128; doi:10.1007/s10875-023-01495-7)

**A**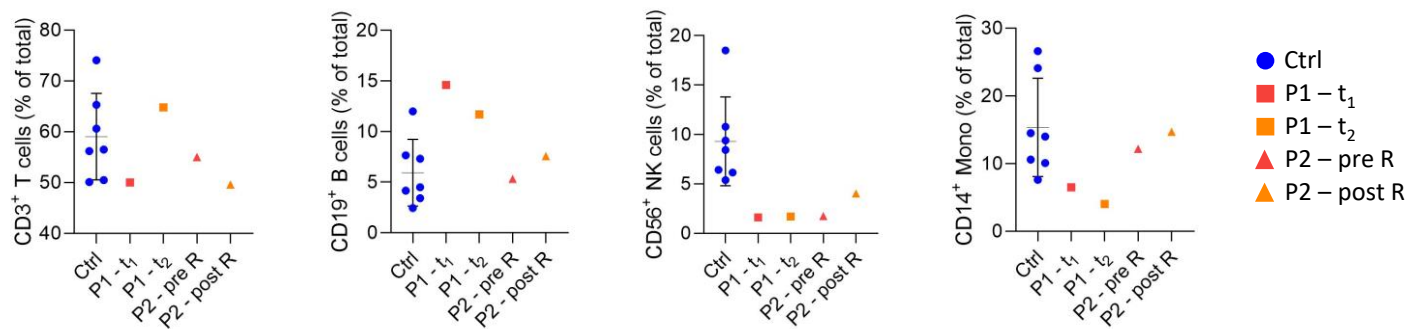**B**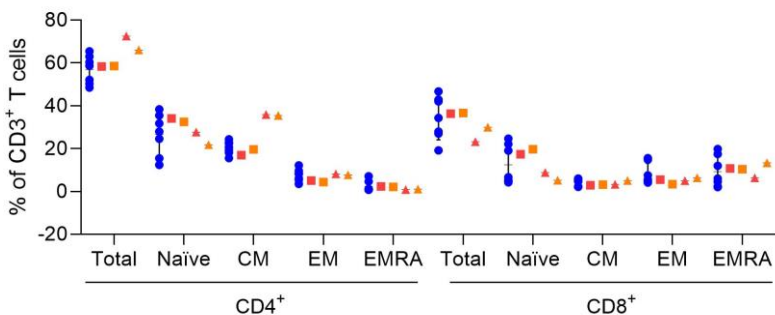**C**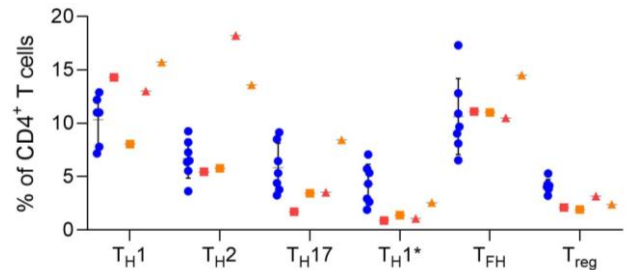**D**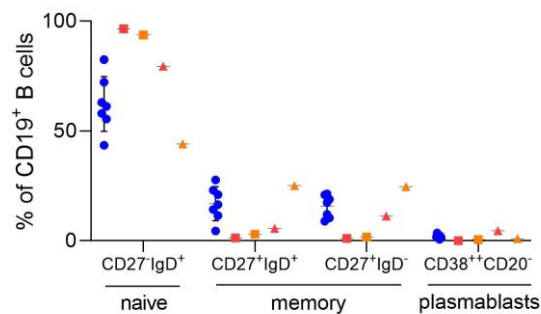**E**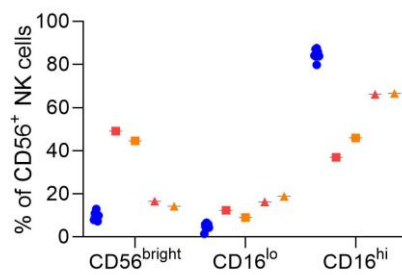**F**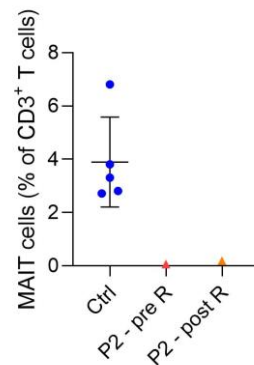

Supplement: Supplementary file 2 — Figure S2 (A-E) Immunophenotype of P1 under ustekinumab treatment, and of P2 before and after 6 months Ruxolitinib treatment by mass cytometry. (F) MAIT cells frequency of P2 before and after 6 months Ruxolitinib treatment by flow cytometry. (PDF 517 kb) [file 10875_2023_1495_MOESM2_ESM.pdf]

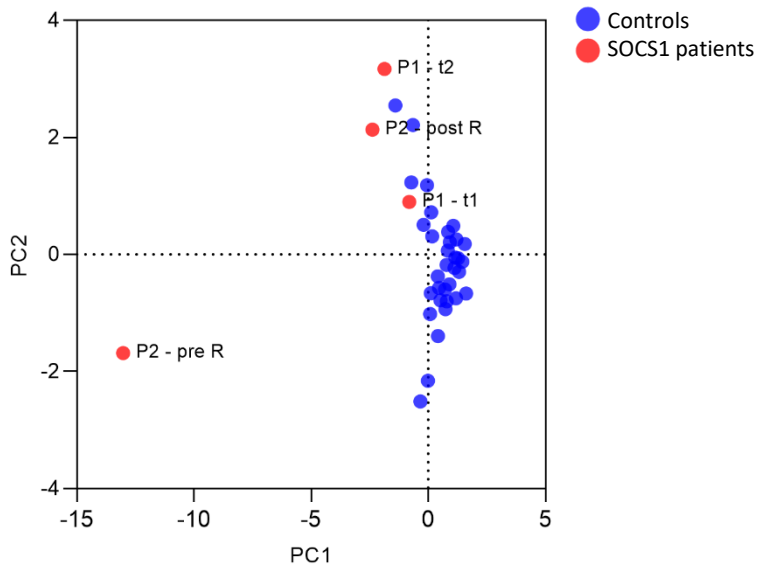

Supplement: Supplementary file 3 — Figure S3 Principal component analyses of cytokines in plasma samples from SOCS1 deficient patients and controls. (PDF 214 kb) [file 10875_2023_1495_MOESM3_ESM.pdf]
