# Supplementary material for: Insights into the expanding intestinal phenotypic spectrum of SOCS1 haploinsufficiency and therapeutic options
Source: J Clin Immunol. 2023 May 9;43(6):1403–13. doi: 10.1007/s10875-023-01495-7 (PMC10354128; doi:10.1007/s10875-023-01495-7)
Supplement: Supplementary file 1 — Figure S1 (A) Sanger-sequencing electropherograms for the affected individuals and carriers. (B) Haplotypes for microsatellite markers are shown in Family 2 and the disease-associated haplotype is boxed in orange. (C) p-STAT5 in EBV-LCLs from a healthy control and P2 stimulated with IL-2 (104 IU/ml for 20 min) with or without Ruxolitinib 200 μM. Data are representative of 2 independent experiments. (PDF 602 kb) [file 10875_2023_1495_MOESM1_ESM.pdf]

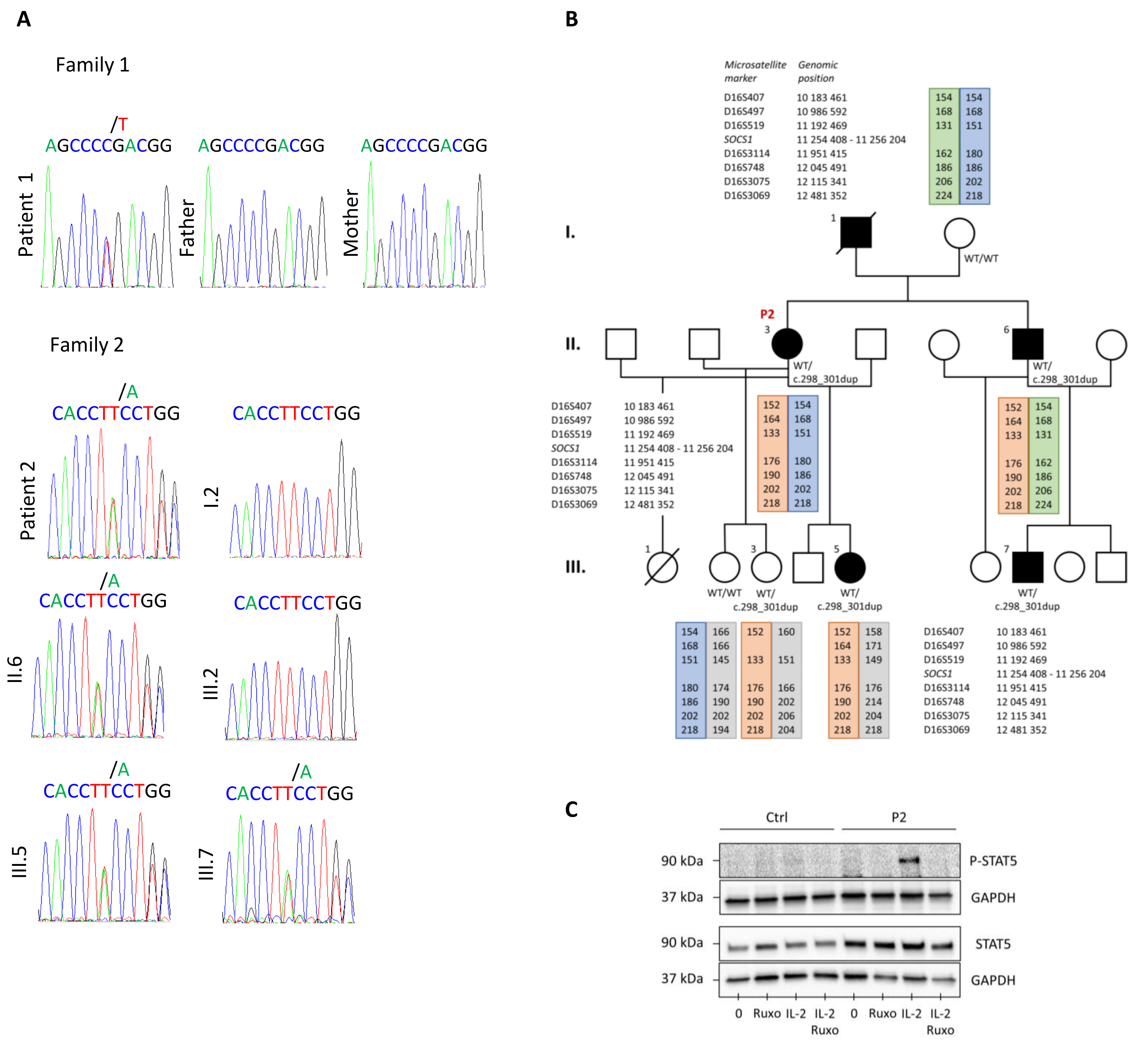
A
Family 1
/T
AGCCCCGACGG
AGCCCCGACGG
AGCCCCGACGG
Patient 1
Father
Mother
Family 2
/A
CACCTTCCTGG
CACCTTCCTGG
Patient 2
I.2
/A
CACCTTCCTGG
CACCTTCCTGG
II.6
III.2
/A
CACCTTCCTGG
/A
CACCTTCCTGG
III.5
III.7
B
Microsatellite marker
Genomic position
D16S407 10 183 461
D16S497 10 986 592
D16S519 11 192 469
SOCS1 11 254 408 - 11 256 204
D16S3114 11 951 415
D16S748 12 045 491
D16S3075 12 115 341
D16S3069 12 481 352
154 154
168 168
131 151
162 180
186 186
206 202
224 218
I.
II.
III.
1
WT/WT
P2
3
6
WT/ c.298_301dup
WT/ c.298_301dup
152 154
164 168
133 151
176 180
190 186
202 202
218 218
152 154
164 168
133 131
176 162
190 186
202 206
218 224
1
3
5
7
WT/WT
WT/ c.298_301dup
WT/ c.298_301dup
WT/ c.298_301dup
154 166
168 166
151 145
180 174
186 190
202 202
218 194
152 160
133 151
176 166
190 202
202 206
218 204
152 158
164 171
133 149
176 176
190 214
202 204
218 218
C
Ctrl
P2
90 kDa
P-STAT5
37 kDa
GAPDH
90 kDa
STAT5
37 kDa
GAPDH
0
Ruxo
IL-2
IL-2 Ruxo
0
Ruxo
IL-2
IL-2 Ruxo
